# Peritoneal Tuberculosis With Septated Fluid Collection: A Case Highlighting the Value of Persistent Diagnostic Efforts

**DOI:** 10.7759/cureus.82377

**Published:** 2025-04-16

**Authors:** Usamah Al-Anbagi, Muayad K Ahmad, Mohamed Mohamedali, Bassem Al Hariri, Memon Noor Illahi, Muhammad Sharif, Abdulqadir J Nashwan

**Affiliations:** 1 Internal Medicine, Hazm Mebaireek General Hospital/Hamad Medical Corporation, Doha, QAT; 2 Medicine, Hamad Medical Corporation, Doha, QAT; 3 Internal Medicine, Hamad Medical Corporation, Doha, QAT; 4 Internal Medicine, Hamad General Hospital, Doha, QAT; 5 Nursing & Midwifery Research, Hamad Medical Corporation, Doha, QAT

**Keywords:** abdominal tuberculosis, anti-tubercular therapy (att), mycobacterium tuberculosis (mtb), peritoneal tuberculosis, polymerase chain reaction (pcr), septated fluid collection

## Abstract

Peritoneal tuberculosis (TB) is an uncommon form of extrapulmonary TB that often presents with nonspecific symptoms, making early diagnosis challenging. Delayed recognition can lead to complications, emphasizing the need for timely identification and treatment. We describe the case of a 25-year-old man who presented with a two-week history of fever and abdominal discomfort. His symptoms included intermittent fever with an evening rise, chills, and unexplained weight loss. He had no prior TB history or known exposures. Initial investigations raised suspicion of a viral or enteric infection, particularly after a positive rhinovirus test. Despite empirical antibiotic therapy, his fever persisted. Imaging revealed complex fluid collections in the abdomen, prompting further evaluation. The first set of microbiological tests, including AFB smears and PCR for *Mycobacterium tuberculosis*, was negative. However, due to high clinical suspicion, repeat PCR testing ultimately confirmed peritoneal TB. Anti-tubercular therapy was initiated, resulting in rapid clinical improvement. This case highlights the diagnostic complexity of peritoneal TB, mainly when initial microbiological tests are inconclusive. Strong clinical suspicion, repeat testing, and targeted imaging play crucial roles in achieving timely diagnosis and initiating appropriate treatment.

## Introduction

Tuberculosis (TB) remains a significant global health challenge, with extrapulmonary forms accounting for a notable proportion of cases. Peritoneal TB is a relatively rare manifestation, yet it poses a diagnostic challenge due to its vague and insidious presentation [1]. Symptoms, such as fever, abdominal pain, ascites, and weight loss, can mimic other conditions, including malignancies and bacterial peritonitis, leading to potential delays in diagnosis and treatment [1]. Abdominal TB can involve various sites, including the peritoneum, stomach, intestines, lymph nodes, liver, pancreas, and perianal area, with the peritoneum, intestines, and lymph nodes being the most frequently affected. Traditional diagnostic methods, including AFB smears and mycobacterial cultures, have limited sensitivity in detecting peritoneal TB [2]. Advanced diagnostic techniques, such as nucleic acid amplification tests (NAATs), cross-sectional imaging, and, in select cases, peritoneal biopsies, can enhance diagnostic accuracy [2]. Early recognition and prompt initiation of anti-tubercular therapy are crucial for improving patient outcomes and preventing complications [3]. In this report, we present a case of peritoneal TB in a young, immunocompetent male who initially exhibited persistent fever and abdominal findings suggestive of an alternative infectious process. The case underscores the importance of maintaining a high index of suspicion for peritoneal TB and highlights the role of repeat testing when initial diagnostic results are inconclusive.

## Case presentation

A 25-year-old gentleman presented to the emergency department with a two-week history of fever and abdominal pain, along with loose stools one week prior and vomiting for the past four days. The fever was moderate-grade, intermittent, associated with chills but no rigor, and exhibited an evening rise in temperature. The abdominal pain was localized to the right hypochondrium, mild in intensity, dull aching, non-radiating, with no specific aggravating or relieving factors. He also reported an occasional dry cough and an unintentional weight loss of 5 kg over the past three weeks.

His history revealed that he had returned from his home country (India) approximately two months before symptom onset. He denied any known sick contacts, significant past medical history, or exposure to animals or unpasteurized milk. He worked as a security guard and shared a room with 14 individuals, none of whom reported similar symptoms.

On examination, he was afebrile, maintaining saturation of 98% on room air, normal blood pressure of 116/80. No coated tongue or rose spots were noted. Abdominal examination revealed right hypochondrial tenderness and generalized guarding, but organomegaly could not be appreciated due to guarding. A respiratory examination revealed inspiratory rhonchi in the left intrascapular area. The remaining clinical examinations were unremarkable. Initial laboratory investigations showed mild leukocytosis with neutrophilia and eosinophilia, hypoalbuminemia, mild coagulopathy, and an elevated CRP (Table 1). Chest x-ray was unremarkable, and a rhinovirus test returned positive. The patient was admitted with a working fever diagnosis with suspicion of viral or protozoal infection, with a differential diagnosis including enteric fever. He was started on empirical ceftriaxone (2 g IV daily), antipyretics, and antiemetics.

**Table 1:** Laboratory investigations WBC: White Blood Cells (Total leukocytes); Hb: Hemoglobin; PLT: Platelet count; BUN: Blood Urea Nitrogen (Serum urea); Cr: Serum Creatinine; K⁺: Serum Potassium; Na⁺: Serum Sodium; Ca²⁺: Serum Calcium; TP: Total Protein; Alb: Albumin; ALT: Alanine Aminotransferase; AST: Aspartate Aminotransferase; ALP: Alkaline Phosphatase; TBil: Total Bilirubin; CRP: C-Reactive Protein; PCT: Procalcitonin; PT: Prothrombin Time; INR: International Normalized Ratio; APTT: Activated Partial Thromboplastin Time.

Parameters	On admission	Day 4	On discharge	Reference values
Total leukocytes	10.4	17.4	11.9	(6.2 x10^3/µL)
Hemoglobin (g/dL)	12.4	10.8	10.7	(13-17 g/dL)
Platelet (x10^3/µL)	262	248	264	(150-410 x10^3/µL)
Serum urea (mmol/L)	6.3	4.8	6.2	(2.5-7.8)
Serum creatinine (µmol/L)	95	82	64	(62-106)
Serum potassium K (mmol/L)	4.9	4.1	4.3	(3.5-5.3)
Serum sodium (mmol/L)	137	128	128	(133-146)
Serum calcium (mmol/L)	2.45	-	-	(2.2-2.6)
Serum total protein (g/L)	75	73	76	(60-80)
Serum albumin (g/L)	28	22	21	(35-50)
ALT (IU/L)	23	54	45	(0-41)
AST (IU/L)	43	102	63	(0-41)
Alkaline phosphatase (U/L)	74	148	256	(40–129)
Serum total bilirubin (mg/dL)	11	11	20	(0-21)
CRP (mg/L)	183	207	204	(0-5 mg/L)
Procalcitonin (ng/mL)	-	26	10.5	(<0.05 ng/mL)
PT (seconds)	18.2	14.7	-	(9.4-12.5 seconds)
INR	1.6	-	1.3	<1
APTT (seconds)	33.8	33.3	-	(25.1-36.5 seconds)

**Figure 1:**
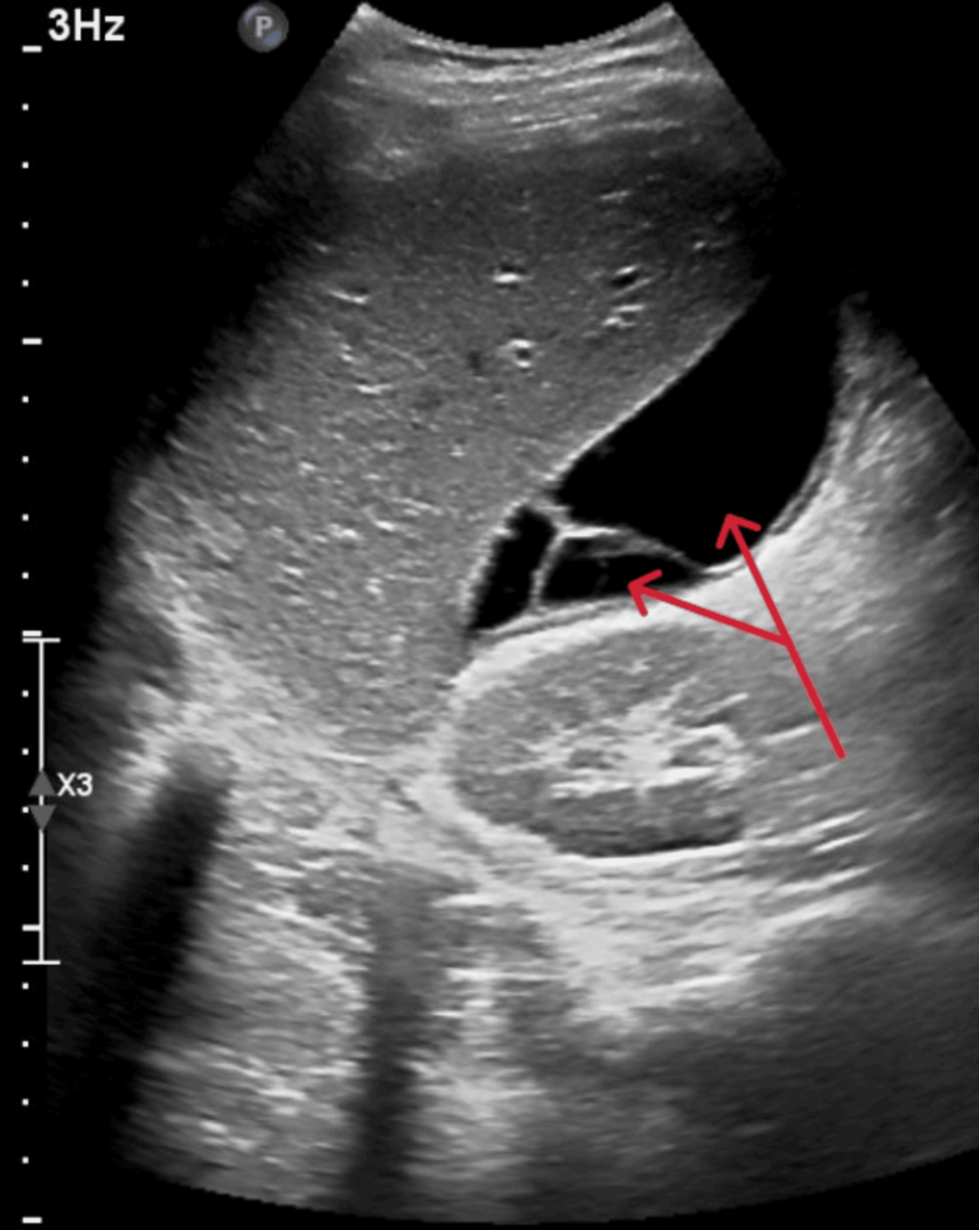
Ultrasound of the abdomen revealed subhepatic collection with septation (red arrows)

Blood cultures, malaria screening, and stool tests for ova and parasites were negative on day 3. Despite treatment, the patient continued to spike fevers, leading to an escalation of antibiotics to piperacillin-tazobactam (4.5 g IV TID) upon approval from the infectious disease specialist. An abdominal ultrasound revealed moderate abdominopelvic ascites with a septated component in the perihepatic space (Figure 1).

**Figure 2:**
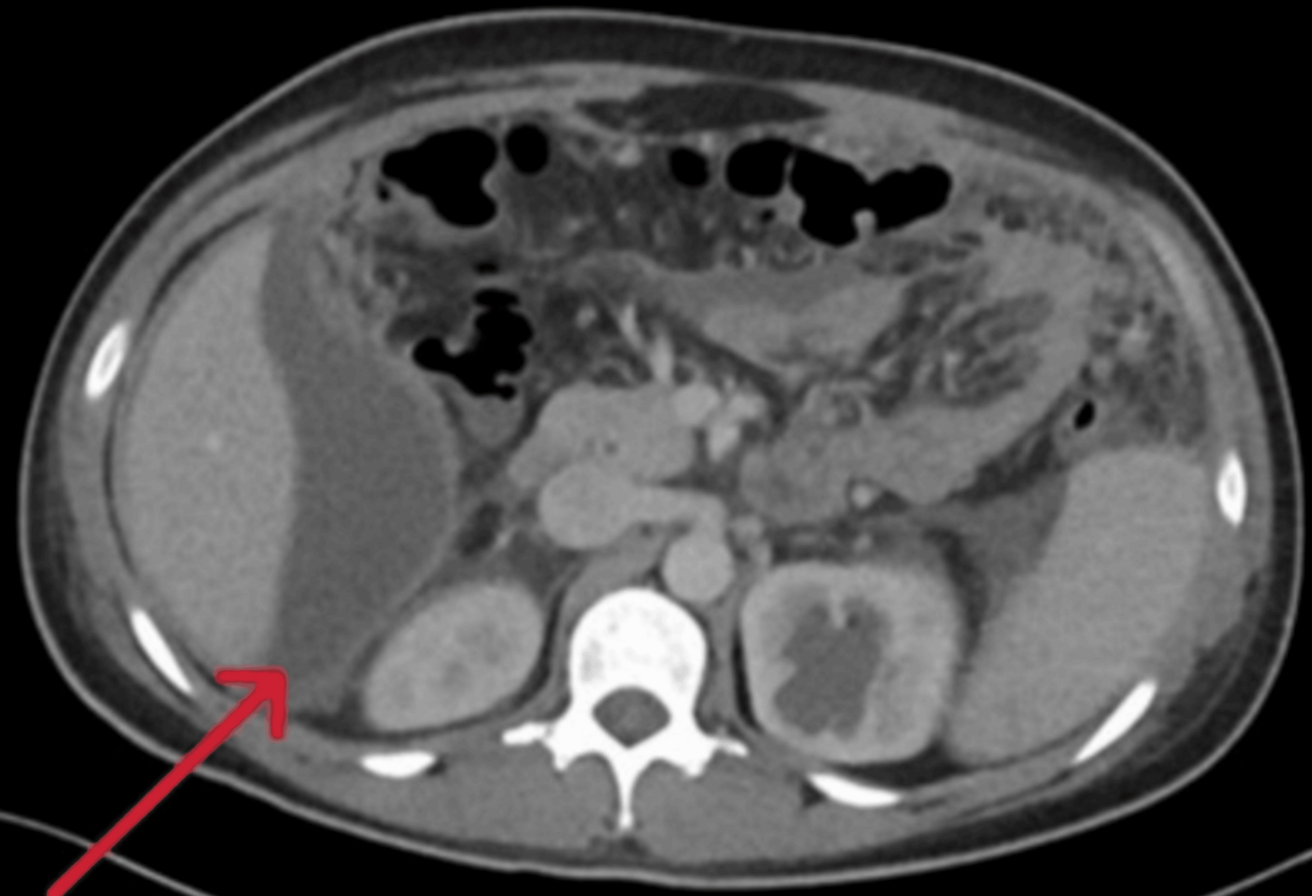
CT scan/axial view revealed subhepatic collection (red arrow)

CT imaging of the abdomen and pelvis showed multiple sizable, interconnected irregular fluid collections with enhancing walls, subhepatic collection measuring 11 × 10 × 4 cm (Figure 2).

**Figure 3:**
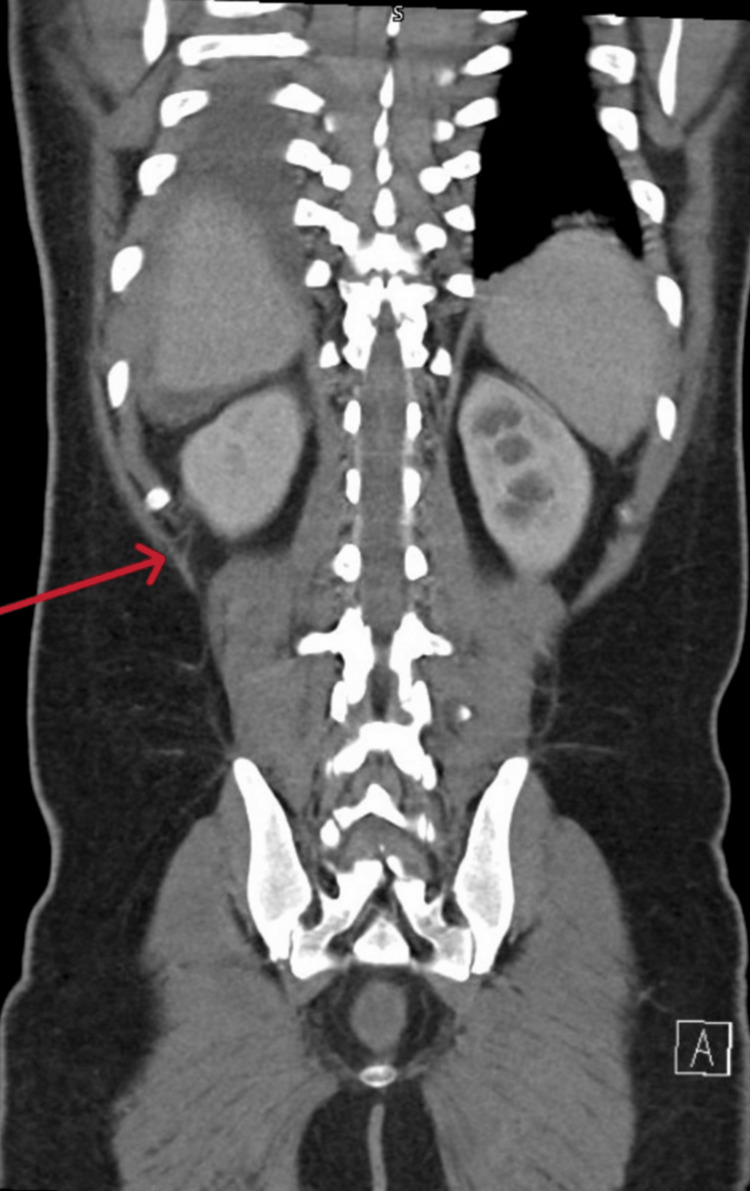
CT scan/coronal view revealed pocket of fluid collection in the right pelvic area with enhancing wall (red arrow)

**Figure 4:**
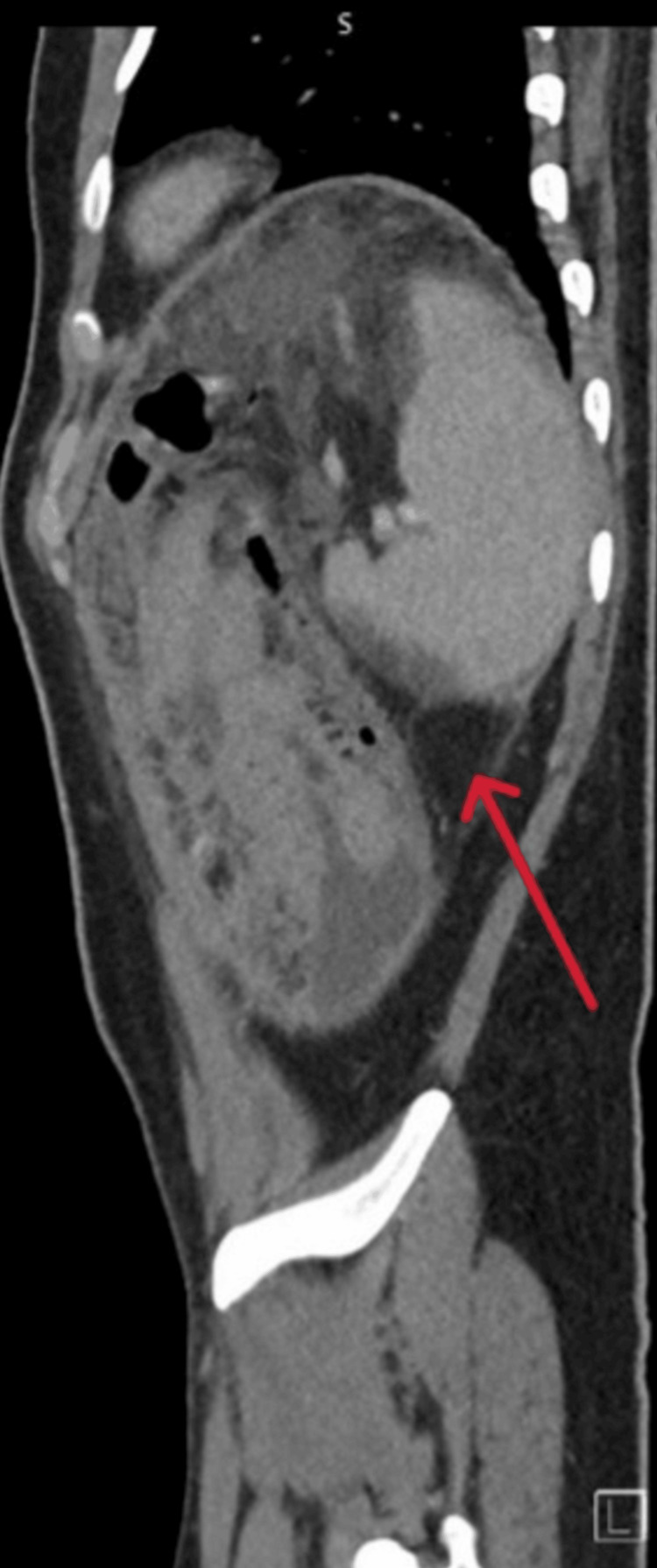
CT scan/sagittal view revealed pocket of fluid collection in the right pelvic area with septation and enhancing wall (red arrow)

Also, the largest measured 15 × 11 × 5 cm in the pelvis (Figures 3, 4).

Given the findings, the interventional radiology team was consulted and decided to insert a pigtail drain for the most extensive collection. Approximately 800 mL of clear fluid was aspirated. Analysis revealed a total cell count of 100, with lymphocytic predominance. The protein level was elevated at 54 g/L, and the serum-ascites albumin gradient (SAAG) was low (<1.1 g/dL), consistent with an exudative ascitic fluid. Initial acid-fast bacilli (AFB) smears from two samples were negative, and polymerase chain reaction (PCR) testing for *Mycobacterium tuberculosis *was also negative (the ascitic fluid was subjected to centrifugation prior to TB testing). However, the patient continued to experience persistent fever, prompting repeat TB testing on the ascitic fluid. Although the repeated AFB smear remained negative, the second PCR test was positive for *M. tuberculosis*, confirming the diagnosis of peritoneal TB with septated ascitic collections.

The patient was started on anti-tubercular therapy (ATT) with a fixed-dose combination of rifampicin (150 mg), isoniazid (75 mg), pyrazinamide (400 mg), ethambutol (275 mg), and pyridoxine (40 mg). Within 24 hours of initiating ATT, his fever completely subsided. He was discharged in stable condition the following day with a scheduled follow-up appointment.

## Discussion

TB peritonitis usually results from the reactivation of latent TB or spread from active pulmonary or miliary TB through hematogenous, lymphatic, or contiguous routes. It can also occur due to ingesting tuberculous mycobacteria (e.g., unpasteurized milk or undercooked meat) or, less commonly, transmural spread from an infected intestine or tuberculous salpingitis (1,4). Peritoneal TB, the most common form, often results from the reactivation of latent TB foci in the peritoneum, initially seeded via hematogenous spread from a primary lung infection [2]. In the U.S. in 2017, 6.2% of extrapulmonary TB cases were peritoneal [3].

It typically arises through the reactivation of latent TB infection, ingestion of tuberculous mycobacteria (e.g., from unpasteurized milk or undercooked meat), or spread from active pulmonary or miliary TB via hematogenous (bloodborne), lymphatic, or contiguous routes (e.g., from adjacent organs, such as the fallopian tubes [1]). Peritoneal TB, the most common form, often results from the reactivation of latent TB foci in the peritoneum, initially seeded via hematogenous spread from a primary lung infection [2]. In the U.S. in 2017, 6.2% of extrapulmonary TB cases were peritoneal [3]. TB peritonitis can also develop from hematogenous spread during active pulmonary or miliary TB, less commonly from transmural spread from an infected intestine, or contiguous spread from tuberculous salpingitis [4]. As the disease progresses, the peritoneum becomes covered with tubercles, leading to protein-rich ascites due to fluid exudation from these lesions. Symptoms may include abdominal pain, distension, fever, weight loss, and ascites, with diagnostic findings often showing mild anemia, elevated sedimentation rates, and sometimes concomitant pulmonary TB [5,6].

The diagnosis of abdominal TB should be considered in patients presenting with symptoms such as fever, weight loss, abdominal pain or distension, ascites, hepatomegaly, diarrhea, abdominal mass, or abnormal liver function tests, particularly if they have risk factors like a history of prior TB infection or exposure, or residence in or travel to TB-endemic regions. A definitive diagnosis can be made by identifying *M. tuberculosis* in peritoneal fluid (in cases of ascites), biopsy specimens (e.g., from the peritoneum, intestine, or liver), or through mycobacterial culture and NAATs [7,8]. Histopathology showing caseating granulomas (with or without AFB) is suggestive but not definitive for TB. Radiographic imaging, particularly computed tomography (CT) with enterography, is recommended to evaluate intestinal and organ involvement, ascites, peritoneal disease, and lymphadenopathy [9].

In tuberculous peritonitis, ascitic fluid is typically straw-colored with a leukocyte count of 150-4,000 cells/mm³, predominantly lymphocytes, and a SAAG <1.1 g/dL. The protein content is usually >3.0 g/dL. An elevated adenosine deaminase (ADA) level (30-39 IU/L) supports the diagnosis but is not definitive. Patients with ascites should undergo paracentesis, with fluid sent for cell count, differential, albumin, protein, Gram stain, ADA level, AFB smear, mycobacterial culture, and NAAT (if available). A peritoneal biopsy is recommended if ascites analysis is inconclusive [10,11]. The sensitivity of AFB smear and mycobacterial culture of ascitic fluid is low, with AFB smear detecting less than 2% of cases and culture less than 20%. Broth culture results may be available in two to three weeks, while solid-phase cultures take several weeks [12]. However, culturing 1 L of ascitic fluid (concentrated by centrifugation) can increase the yield of mycobacterial culture to up to 83%. The role of NAAT, such as PCR, for diagnosing tuberculous peritonitis is not well established. However, one review of 11 abdominal TB cases reported positive PCR for *M. tuberculosis* in all cases [13].

Patients with abdominal TB should be treated with anti-tuberculous therapy, following the same regimen as for pulmonary TB [14,15]. In cases where a definitive diagnosis cannot be established but there is a high clinical suspicion based on symptoms, epidemiologic factors, and supportive findings (e.g., elevated ascitic fluid ADA or consistent histology), an empiric trial of anti-tuberculous therapy is reasonable [16]. Fever typically resolves within one week of starting treatment, and patients with ascites or tuberculous enteritis usually show improvement within weeks. However, healing can lead to stricture formation due to scar tissue, particularly in the intestines, and surgical intervention may be required for complications such as perforation, abscess, fistula, bleeding, or high-grade obstruction. In a study of 106 patients with stricturing intestinal TB, only 25% experienced stricture resolution after anti-tuberculous therapy [17]. If there is no clinical response within four to eight weeks, alternative diagnoses like Crohn's disease, lymphoma, or malignancy should be considered [18].

Our case highlights the diagnostic challenges associated with peritoneal TB, particularly when initial microbiological tests yield negative results. Despite classic symptoms, including fever, abdominal pain, and weight loss, the diagnosis was initially unclear due to an atypical presentation and a positive rhinovirus test, leading to a preliminary consideration of viral or enteric infections. The persistence of fever despite broad-spectrum antibiotics prompted further evaluation, ultimately revealing septated ascitic collections. The initial AFB smear and PCR tests on ascitic fluid were negative, underscoring the limitations of single-sample testing. However, a repeat NAAT, which offers higher sensitivity and specificity than conventional PCR, confirmed the presence of *M. tuberculosis* in the ascitic fluid [19]. This enabled the timely initiation of anti-tubercular therapy, leading to rapid clinical improvement. This case emphasizes the need for a high index of suspicion for peritoneal TB in patients with unexplained fever and ascitic collections, especially in endemic regions. It highlights the importance of repeated testing in cases with inconclusive initial results.

## Conclusions

This case underscores the diagnostic challenges of peritoneal TB, particularly when initial microbiological tests yield negative results. The patient’s persistent fever and abdominal symptoms, coupled with inconclusive early investigations, highlight the need for a high index of suspicion and the role of repeat testing in confirming the diagnosis. The successful identification of *M. tuberculosis* on repeat PCR testing and the patient’s rapid clinical improvement following anti-tubercular therapy emphasize the importance of early recognition and timely intervention. Clinicians should remain vigilant for peritoneal TB in patients with unexplained ascitic collections and fever, particularly in endemic regions, and should consider repeat testing when initial diagnostic results are inconclusive.
